# Inter-Observer and Intra-Observer Variations in the Assessment of Epithelial Dysplasia in Oral Lichenoid Diseases

**DOI:** 10.3390/dermatopathology8020013

**Published:** 2021-04-01

**Authors:** Marwa Zohdy, Simone Cazzaniga, Helga Nievergelt, Roland Blum, Valérie G. A. Suter, Laurence Feldmeyer, Helmut Beltraminelli

**Affiliations:** 1 Department of Dermatology, Inselspital Bern University Hospital, University of Bern, CH-3010 Bern, Switzerland; marwazohdy@mans.edu.eg (M.Z.); cazzaniga_81@hotmail.it (S.C.); helga.nievergelt@insel.ch (H.N.); roland.blum@insel.ch (R.B.); helmut.beltraminelli@eoc.ch (H.B.); 2 Department of Dermatology, Andrology and STDs, Mansoura University Hospitals, Mansoura University, Mansoura 35516, Egypt; 3 Centro Studi GISED, 24121 Bergamo, Italy; 4 Department of Oral Surgery and Stomatology, School of Dental Medicine, University of Bern, CH-3010 Bern, Switzerland; valerie.suter@zmk.unibe.ch

**Keywords:** oral lichen planus, oral lichenoid lesion, oral lichenoid disease, dysplasia, inter-observer, intra-observer

## Abstract

Oral lichen planus (OLP) and oral lichenoid lesions (OLL) can both present with histological dysplasia. Despite the presence of WHO-defined criteria for the evaluation of epithelial dysplasia, its assessment is frequently subjective (inter-observer variability). The lack of reproducibility in the evaluation of dysplasia is even more complex in the presence of a lichenoid inflammation. We evaluated dysplasia in 112 oral biopsies with lichenoid inflammation in order to study the inter-observer and the intra-observer variability.

## 1. Introduction

Oral lichen planus (OLP) is the oral manifestation of lichen planus, a common inflammatory dermatologic condition involving the skin, hair follicles, nails, and mucous membranes [1]. The diagnosis is based on the combination of clinical and histopathological criteria; the latter include a band-like subepithelial inflammation with the hydropic degeneration of the basal keratinocytes. Twenty percent of patients with an OLP also have skin lesions [2], and these are very important to distinguish OLP from oral lichenoid lesions (OLL). OLL are a group of diseases characterized clinically by a stomatitis, and histologically by a lichenoid reaction similar to OLP, but typically associated with an attributable etiology [1]: allergic lichenoid contact mucositis, oral lichenoid drug reactions, lichenoid graft versus host disease, and other lichenoid reactions [3,4]. OLP and OLL can present clinically with plaque formation, atrophy, erosions or bullae.

Despite the presence of WHO-defined criteria to evaluate epithelial dysplasia—such as the presence of several architectural and cellular changes—their assessment is frequently subjective (inter-observer variability), and is therefore inconsistent [5,6,7,8,9]. Moreover, the significance of these alterations may also receive a subjective interpretation from different experts: some see them as reactive changes, other as a true sign of (pre-)malignancy [5,6]. There is also an intra-observer variability, in which the same pathologist may have a different opinion about the presence of dysplasia assessed at two different times [7]. The lack of reproducibility in the evaluation of dysplasia is even more complex in the presence of a lichenoid inflammation, when the keratinocytes show reactive alterations which may appear as dysplastic changes. However, we did not find any study about intra- and inter-observer variability in the specific assessment of dysplasia in OLP in comparison to OLL. We therefore evaluated dysplasia in 112 oral biopsies with lichenoid inflammation in order to study the inter-observer and the intra-observer variability.

## 2. Materials and Methods

In a monocentric, retrospective, interdisciplinary study (dermatology, dental medicine, and dermatopathology), four board-certified dermatopathologists who routinely sign out oral mucosal biopsies for the evaluation of dysplasia evaluated the grade of epithelial dysplasia in 112 biopsies with an oral lichenoid inflammation (all were patients with a confirmed diagnosis of OLP or OLL). All of the consecutive cases were retrieved from the archives of our tertiary referral center during the 34-month study period. The clinical and histological criteria to differentiate OLP from OLL were described in a previous study [10]. Each of the four dermatopathologists evaluated, on one selected slide, the grade of dysplasia (0 = no dysplasia, 1 = mild, 2 = moderate, 3 = severe dysplasia = carcinoma in situ), without information about the original histopathological assessment, clinical data, or patient identity. The dysplasia was diagnosed and graded according to the latest WHO classification, and included the changes in the basal (grade 1) and suprabasal (grade 2) epithelium, and in the entire thickness of the epithelium in grade 3. The examined criteria were architectural and cellular changes [11,12]. The same histological evaluation of dysplasia was repeated, blinded, three months later.

We statistically assessed the intra- and the inter-observer variation of this evaluation according to Cohen’s kappa, with quadratic weights to account for the ordered scores. For the inter-observer reliability, the weighted kappa was computed based on two-way random, single measures intra-class correlation. The kappa was reported along with its 95% confidence interval (CI), and can be interpreted as follows: <0, poor; 0–0.20, slight; 0.21–0.40, fair; 0.41–0.60, moderate; 0.61–0.80, substantial; and >0.80, almost perfect agreement. The comparison between the OLL and OLP patients was based on the median values of all of the examiners’ assessments, and was tested by using a Mann–Whitney U test. All of the tests were considered significant at a *p* value < 0.05. The analyses were performed in SPSS v.26 (IBM Corp. Armonk, NY, USA).

## 3. Results

**Figure 1:**
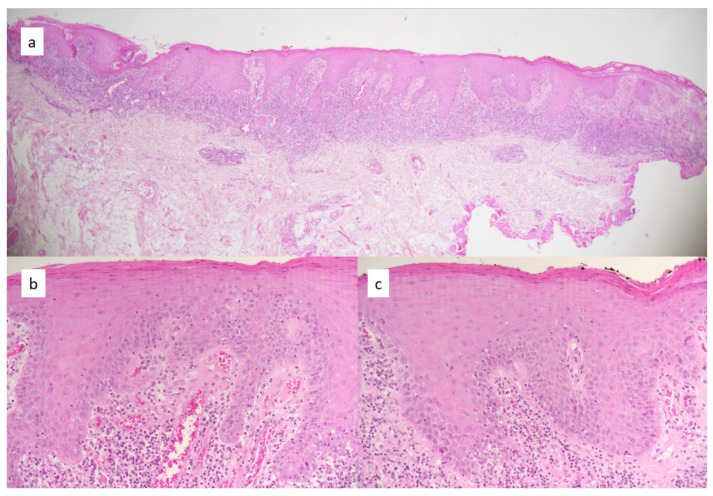
Pictures of an OLL, with magnification on different zones of the same lesion. (**a**) Overview, (**b**) detail of an area with no atypia, (**c**) detail of an area with the presence of atypical keratinocytes. The dysplasia grading among the four experts for the whole slide was as follows (first evaluation/second evaluation at 3 months. Expert 1: dysplasia grading 0/2; Expert 2: dysplasia grading 1/2; Expert 3: dysplasia grading 2/2; Expert 4: dysplasia grading 2/3. Dysplasia grading: 0 = none, 1 = mild, 2 = moderate, 3 = severe.

In a previous study, we clinically identified 84 patients with the criteria of OLP, and 28 with OLL [10]. We found a great variability of the interpretation of dysplasia, with a low inter-observer reliability among the four examiners. The kappa was rated very low, measuring 0.05 (95% CI: 0.01, 0.13) and 0.11 (0, 0.25), respectively, in the two repeated assessments. The intra-observer reliability varied among the examiners: the kappa was 0.34 (0.14, 0.54) and 0.36 (0.19, 0.53) (fair agreement) for two examiners, 0.52 (0.30, 0.75) for another examiner (moderate), and 0.65 (0.50, 0.80) for the last examiner (substantial). Despite the variability of the assessment of dysplasia between the experts, the degree of dysplasia was significantly higher in the OLL than the OLP cases (*p* = 0.03) (Figure 1 and Table 1).

Analyzing the amount of variability in the evaluation of the grade of dysplasia in OLP and OLL, we found a significant inconsistency among the experts (inter-observer variations), and in three of four experts a significant intra-observer discrepancy (poor to moderate agreement) when examining the same slide at two different times. One of the four experts consistently recorded a higher dysplasia grade compared to the other three, and this persisted in the second evaluation. Experts A, B, and C never (but in one case) evaluated dysplasia as grade 3, whereas expert D evaluated dysplasia as grade 3 in 24 (29%) of the cases. Moreover, experts A, B, and C did not record any dysplasia in 66–88% of the cases, but the expert D found some dysplasia in all of the cases. Furthermore, experts A, B, and C found a moderate dysplasia (grade 2) in 1–4% of the cases, but expert D saw it in 61% of the cases (Table 2). The intra-observer variability did not vary according to the number of years in practice. Despite these inter-observer variations, the degree of dysplasia was significantly higher in the OLL than the OLP cases, as was previously demonstrated in other studies [13].

## 4. Discussion

Both OLP and OLL may histologically show some morphological reactive changes of the keratinocytes, which may be interpreted as dysplasia, but this interpretation is a controversial, debated issue among the experts. Krutchkoff and Eisenberg [14] proposed a list of histologic criteria for the so called ‘lichenoid dysplasia’, a lesion with the histopathologic characteristics of OLP with additional presence of dysplasia. This term was not adopted by other authors; in fact, according to van der Meij and van der Waal, the absence of epithelial dysplasia should be required for the histological diagnosis of OLP [5]. Furthermore, cases with a true epithelial dysplasia and an additional lichenoid reaction (a reactive lichenoid inflammation due to the neoplasia) may bring some additional confusion [5]. Finally, from the pathophysiological point of view, it is well known that in a cytotoxic tissue reaction like a lichenoid dermatitis/stomatitis, some epithelial cells may appear atypical/dysplastic [15].

In the literature, there are some examples of inter-observer and/or intra-observer variation in the histological evaluation of oral dysplasia [6,7,8,9,16,17]. Most authors conclude that the potential bias induced by the subjectivity of the examiner can only be reduced with the help of clearer diagnostic criteria, and with a systematic histological evaluation. An interesting solution is the use of a binary system with low/high risk lesions, as it is applied in the evaluation of female and male intra-epithelial neoplasia of the genital area. Küffer and Lombardi 2002 proposed the term of oral intraepithelial neoplasia, with a grading into low and high grade [18]. Taking into consideration the results of this study, we strongly encourage other specialists dealing with the evaluation of dysplasia in oral lichenoid diseases to use a binary system. Ideally, all of the specialists dealing with patients with an oral lichenoid disease should discuss together the therapeutic consequences of the currently-used histological analysis [8,9].

## 5. Conclusions

In conclusion, we demonstrated that, in OLL, there is significantly more epithelial dysplasia than in OLP. We also showed that the grading of epithelial dysplasia in OLP/OLL is partly subjective, showing both significant inter-observer and intra-observer variations. In order to avoid this confusing variability, we strongly encourage specialists to use a binary system.

## Figures and Tables

**Table 1:** Intra-rater reliability of the dysplasia assessment for each rater, and overall.

First Assessment		Second Assessment								Weighted Kappa * (95% CI)
		None		I		II		III		
		N	Row%	N	Row%	N	Row%	N	Row%	
Rater: Pathologist 1	None	65	73.0%	24	27.0%	0	0.0%	0	0.0%	0.36 (0.19, 0.53)
	I	7	31.8%	14	63.6%	1	4.5%	0	0.0%	
	II	0	0.0%	1	100.0%	0	0.0%	0	0.0%	
	III	0	0.0%	0	0.0%	0	0.0%	0	0.0%	
Rater: Pathologist 2	None	68	75.6%	15	16.7%	7	7.8%	0	0.0%	0.34 (0.14, 0.54)
	I	7	35.0%	9	45.0%	4	20.0%	0	0.0%	
	II	0	0.0%	1	50.0%	0	0.0%	1	50.0%	
	III	0	0.0%	0	0.0%	0	0.0%	0	0.0%	
Rater: Pathologist 3	None	84	94.4%	5	5.6%	0	0.0%	0	0.0%	0.52 (0.30, 0.75)
	I	14	73.7%	2	10.5%	3	15.8%	0	0.0%	
	II	1	25.0%	1	25.0%	1	25.0%	1	25.0%	
	III	0	0.0%	0	0.0%	0	0.0%	0	0.0%	
Rater: Pathologist 4	None	0	0.0%	0	0.0%	0	0.0%	0	0.0%	0.65 (0.50, 0.80)
	I	0	0.0%	7	38.9%	10	55.6%	1	5.6%	
	II	0	0.0%	4	5.9%	57	83.8%	7	10.3%	
	III	0	0.0%	1	3.8%	1	3.8%	24	92.3%	

CI: confidence interval; * weighted kappa with quadratic weights.

**Table 2:** Inter-rater reliability of the dysplasia assessment.

Dysplasia Assessement		Path. 1		Path. 2		Path. 3		Path. 4		Weighted Kappa * (95% CI)	Weighted Kappa (*w*/*o* Path. 4) (95% CI)
		N	Col%	N	Col%	N	Col%	N	Col%		
First dysplasia assessment	None	89	79.5%	90	80.4%	89	79.5%	0	0.0%	0.05 (−0.005, 0.13)	0.13 (0.02, 0.25)
	I	22	19.6%	20	17.9%	19	17.0%	18	16.1%		
	II	1	0.9%	2	1.8%	4	3.6%	68	60.7%		
	III	0	0.0%	0	0.0%	0	0.0%	26	23.2%		
Second dysplasia assessment	None	72	64.3%	75	67.0%	99	88.4%	0	0.0%	0.11 (0.002, 0.25)	0.37 (0.25, 0.49)
	I	39	34.8%	25	22.3%	8	7.1%	12	10.7%		
	II	1	0.9%	11	9.8%	4	3.6%	68	60.7%		
	III	0	0.0%	1	0.9%	1	0.9%	32	28.6%		

* Weighted kappa with quadratic weights computed based on two-way random, single measures, absolute agreement intra-class correlation. Path.: pathologist.
